# Qualitative robustness of utility-based risk measures

**DOI:** 10.1007/s10479-022-04885-z

**Published:** 2022-08-08

**Authors:** Pablo Koch-Medina, Cosimo Munari

**Affiliations:** Center for Finance and Insurance and Swiss Finance Institute, University of Zurich, Zurich, Switzerland

**Keywords:** Risk measures, Utility functions, Qualitative robustness, Continuity

## Abstract

We contribute to the literature on statistical robustness of risk measures by computing the index of qualitative robustness for risk measures based on utility functions. This problem is intimately related to finding the natural domain of finiteness and continuity of such risk measures.

## Introduction

Risk measures were introduced in Artzner et al. (1999) as a means of quantifying the amount of capital a financial institution needs to raise and invest in an “eligible” asset so as to pass a pre-specified capital adequacy test. The bulk of the literature has focused on cash-additive risk measures, i.e., risk measures for which the eligible asset is cash (the risk-free asset when the interest rate is zero). In this note we deal exclusively with cash-additive risk measures. We refer to Farkas et al. (2014) for a comprehensive treatment of risk measures with respect to a general eligible asset and to Artzner et al. (2009), Föllmer and Schied (2002), and Farkas et al. (2015) for extensions to multiple eligible assets. Mathematically, a risk measure is modelled as a functional defined on a suitable space of random variables, which represent, e.g., the profit-and-loss profile of a bank or insurance firm. In all existing capital adequacy regimes, risk measures are law invariant, i.e., they depend only on the probability distribution of the profit-and-loss profile. In practice, one can never determine this probability distribution with certainty and can only infer it as the result of an estimation procedure based on historical observations. This procedure is, by necessity, susceptible to misestimation. A natural and critical problem is therefore that of determining the degree of sensitivity or robustness of a risk measure with respect to misestimations of the input data. From a mathematical perspective, this requires specifying a notion of “distance” for probability distributions and studying the continuity properties of the risk measure with respect to this distance.

In a broader context than that of risk measures, the notion of qualitative robustness was introduced in Hampel (1971). This notion essentially boils down to continuity with respect to the so-called Lévy distance. Relying on Hampel’s work, Cont et al. (2010) studied the qualitative robustness properties of cash-additive risk measures. However, as pointed out in Krätschmer et al. (2012) and reinforced in Krätschmer et al. (2014), this concept of qualitative robustness may not be ideally suited for risk measures. Indeed, the capital requirements of two financial institutions whose profit-and-loss distributions have widely different “tails” should, intuitively speaking, be significantly different. However, this may not be the case when using a risk measure that is robust according to Hampel’s definition because two distributions may possess different “tail” behaviour and yet be “close” to each other with respect to the Lévy distance. To ensure a more appropriate tail sensitivity, a refined notion of qualitative robustness was introduced in Krätschmer et al. (2012) and applied to risk measures in Krätschmer et al. (2014). In these papers, the degree of qualitative robustness on $L^p$ spaces is quantified by the so-called *index of qualitative robustness*. As shown in Koch-Medina and Munari (2014), determining the index of qualitative robustness is equivalent to identifying the largest $L^p$ space to which the underlying risk measures can be extended without losing finiteness and continuity. The index of qualitative robustness has been computed for a number of classes of risk measures, including distortion risk measures and max-correlation risk measures; see Koch-Medina and Munari (2014) and Krätschmer et al. (2014). In this note we focus on utility-based risk measures. This class of risk measures includes some well-studied examples like the entropic risk measure and has been thoroughly investigated in the literature; see. e.g., Arai (2010), Armenti et al. (2018), Föllmer and Schied (2002), Föllmer and Knispel (2011), Geissel et al. (2018), Weber (2006). The index of qualitative robustness for utility-based risk measures has been characterized in Koch-Medina and Munari (2014) under the assumption that the underlying utility functions are bounded from above. This requirement was postulated for technical reasons and does not cover some relevant examples considered in the literature. The goal of this note is to provide an explicit formula for the index of qualitative robustness for general utility-based risk measures. Besides delivering a general result, the approach pursued here is more direct and allows us to derive, along the way, new results on finiteness and continuity of expected utility functionals that are of independent interest. The main result is Theorem 5.6, which shows that the index of qualitative robustness for a utility-based risk measure associated with a utility function *u* is explicitly given by

$$\begin{aligned} \inf \left\{ p\in [1,\infty ) \,; \ \limsup _{x\rightarrow \infty }\frac{x^p}{u(-x)}<0\right\} ^{-1}. \end{aligned}$$

This formula is simple to compute for all standard utility functions encountered in the literature and shows that the index of qualitative robustness only depends on the “tail” behavior of *u*, i.e., the asymptotic behavior at $-\infty$. It also shows, a posteriori, that the restrictive assumption in Koch-Medina and Munari (2014) was not necessary.

The note is organized as follows. After reviewing some fundamental notions from the theory of risk measures in Sect. 2, we discuss extensions of risk measures preserving finiteness and continuity in Sect. 3 and their statistical robustness in Sect. 4. Utility-based risk measures are studied in Sect. 5, which contains our main results.

## Risk measures

The mathematical setting is the following. Throughout the note, we fix a nonatomic probability space $(\Omega ,{\mathcal {F}},{\mathbb {P}})$. All “almost-sure” notions are to be understood with respect to ${\mathbb {P}}$. The standard Lebesgue spaces are denoted by $L^p$ for $p\in [1,\infty ]$ and are equipped with their standard Banach lattice structure with canonical norm denoted by $\Vert \cdot \Vert _p$. Recall that the elements of $L^p$ are equivalence classes of Borel measurable functions $X:\Omega \rightarrow {\mathbb {R}}$ with respect to almost-sure equality. As usual, we do not explicitly distinguish between an equivalence class and any of its representatives. The elements of ${\mathbb {R}}$ are identified with (equivalence classes of) random variables that are almost-surely constant. All equalities and inequalities have to be understood in the almost-sure sense. The closure of a set ${\mathcal {A}}\subset L^p$ is denoted by ${\mathrm{cl}}_p({\mathcal {A}})$. Finally, for every random variable *X* we denote by ${\mathbb {P}}_X$ its probability law.

Assuming that the profit and loss of a financial institution is described by a random variable $X\in L^p$, with $p\in [1,\infty ]$, we consider risk measures on $L^p$. The primitive notions in the theory of risk measures are the acceptance set, encapsulating the criterion for deeming a financial institution to be adequately capitalized, and the eligible asset, specifying how capital that is raised needs to be invested, which throughout this note will be assumed to be cash. The risk measure then corresponds to the minimum amount of capital that needs to be raised and held in cash so as to ensure acceptability.

### Definition 2.1

Let $p\in [1,\infty ]$. A set ${\mathcal {A}}\subset L^p$ is called an *acceptance set* if it is nonempty, strictly contained in $L^p$, and monotone in the sense that for all $X,Y\in L^p$

$$\begin{aligned} X\in {\mathcal {A}}, \ Y\ge X \ \implies \ Y\in {\mathcal {A}}. \end{aligned}$$

The *(cash-additive) risk measure* associated with ${\mathcal {A}}$ is the map $\rho _{\mathcal {A}}:L^p\rightarrow [-\infty ,\infty ]$ defined by

$$\begin{aligned} \rho _{\mathcal {A}}(X) := \inf \{m\in {\mathbb {R}}\,; \ X+m\in {\mathcal {A}}\}. \end{aligned}$$

The main results of this note are stated for acceptance sets that are convex and law invariant.

### Definition 2.2

Let $p\in [1,\infty ]$. An acceptance set ${\mathcal {A}}\subset L^p$ is called:

1. *Convex* if $\lambda X+(1-\lambda )Y\in {\mathcal {A}}$ for all $X,Y\in {\mathcal {A}}$ and $\lambda \in [0,1]$.
2. *Law invariant* if $Y\in {\mathcal {A}}$ for all $X\in {\mathcal {A}}$ and $Y\in L^p$ with ${\mathbb {P}}_X={\mathbb {P}}_Y$.

The following properties of a cash-additive risk measure are well known and easy to establish.

### Proposition 2.3

Let $p\in [1,\infty ]$. For an acceptance set ${\mathcal {A}}\subset L^p$ the following statements hold:

(i) $\rho _{\mathcal {A}}$ is cash additive, i.e., for all $X\in L^p$ and $m\in {\mathbb {R}}$
  $$\begin{aligned} \rho _{\mathcal {A}}(X+m)=\rho _{\mathcal {A}}(X)-m. \end{aligned}$$
(ii) $\rho _{\mathcal {A}}$ is nonincreasing, i.e., for all $X,Y\in L^p$
  $$\begin{aligned} Y\ge X \ \implies \ \rho _{\mathcal {A}}(Y)\le \rho _{\mathcal {A}}(X). \end{aligned}$$
(iii) If ${\mathcal {A}}$ is convex, then $\rho _{\mathcal {A}}$ is convex, i.e., for all $X,Y\in L^p$ and $\lambda \in [0,1]$
  $$\begin{aligned} \rho _{\mathcal {A}}(\lambda X+(1-\lambda )Y) \le \lambda \rho _{\mathcal {A}}(X)+(1-\lambda )\rho _{\mathcal {A}}(Y). \end{aligned}$$
(iv) If ${\mathcal {A}}$ is law invariant, then $\rho _{\mathcal {A}}$ is law invariant, i.e., for all $X,Y\in L^p$
  $$\begin{aligned} {\mathbb {P}}_X={\mathbb {P}}_Y \ \implies \ \rho _{\mathcal {A}}(X)=\rho _{\mathcal {A}}(Y). \end{aligned}$$

## Finiteness and continuity

The quest for the most natural model space for a given class of risk measures, i.e., the “largest space” to which a risk measure can be extended preserving certain key properties, has been the subject of extensive research; see, e.g., Bellini et al. (2021), Delbaen (2002), Delbaen (2009), Filipović and Svindland (2012), Koch-Medina and Munari (2014), Liebrich and Svindland (2017), Pichler (2013). It is well known that any cash-additive risk measure defined on $L^\infty$ is finite valued and continuous, in fact Lipschitz continuous; see, e.g., Föllmer and Schied (2016, Lemma 4.3). It is also well known that a cash-additive risk measure defined on $L^\infty$ that is convex and law invariant can always be uniquely extended to a cash-additive risk measure on $L^1$ that is convex, law invariant, and lower semicontinuous; see, e.g., Bellini et al. (2021) and Filipović and Svindland (2012). Clearly, this also ensures the existence of law-invariant, convex, and lower semicontinuous extensions to any intermediate space $L^p$. It is natural to ask whether these extensions are also finite valued. This is, however, not necessarily the case (see, e.g., Farkas et al. (2014)), which is not surprising given that, for a convex and monotone functional, finiteness is such a strong property that it automatically implies continuity.

### Proposition 3.1

(Borwein (1987, Corollary 2.4), Ruszczyński and Shapiro (2006, Proposition 3.1)). Let $p\in [1,\infty ]$ and let $\rho :L^p\rightarrow [-\infty ,\infty ]$ be convex and nonincreasing. If $\rho$ is finite valued, then it is continuous.

The preceding discussion leads to defining the following index of finiteness, which identifies the largest $L^p$ space where cash-additive extensions of cash-additive risk measures preserve finiteness. Note that the index is finite precisely when a finite extension to some intermediate $L^p$ space exists.

### Definition 3.2

Let ${\mathcal {A}}\subset L^\infty$ be an acceptance set. The *index of finiteness* of $\rho _{\mathcal {A}}$ is

$$\begin{aligned}&{\mathrm{fin}}(\rho _{\mathcal {A}}) := \inf \{p\in [1,\infty ) \,; \\&\quad \ \rho _{\mathcal {A}} \text{ can } \text{ be } \text{ extended } \text{ to } \text{ a } \text{ finite-valued } \text{ cash-additive } \text{ risk } \text{ measure } \text{ on } L^p\}. \end{aligned}$$

The next result records a characterization of the index of finiteness for cash-additive risk measures associated with law-invariant and convex acceptance sets.

### Theorem 3.3

(Koch-Medina and Munari (2014, Theorem 4.3)). Let ${\mathcal {A}}\subset L^\infty$ be a law-invariant convex acceptance set and take $p\in [1,\infty )$. The following statements are equivalent:

(i) $\rho _{\mathcal {A}}$ can be extended to a finite-valued (hence, continuous) cash-additive risk measure on $L^p$.
(ii) ${\mathrm{cl}}_p({\mathcal {A}})$ has nonempty interior in $L^p$.

In this case, the extension is unique and given by $\rho _{{\mathrm{cl}}_p({\mathcal {A}})}$. Moreover,

$$\begin{aligned} {\mathrm{fin}}(\rho _{\mathcal {A}}) = \inf \{p\in [1,\infty ) \,; \ {\mathrm{cl}}_p({\mathcal {A}}) \text{ has } \text{ nonempty } \text{ interior } \text{ in } L^p\}. \end{aligned}$$

## Qualitative robustness

In practice, law-invariant risk measures are computed by means of statistical estimation. A standard approach in many applications is to use historical estimators, which are plug-in estimators based on the empirical distribution of historical observations. The standard robustness concept associated with historical estimators is based on the Lévy metric and goes back to Hampel (1971). Let ${\mathcal {M}}$ be the set of (Borel) probability measures over ${\mathbb {R}}$ and define

$$\begin{aligned} {\mathcal {M}}^\infty := \{\mu \in {\mathcal {M}}\,; \ \mu ={\mathbb {P}}_X \text{ for } \text{ some } X\in L^\infty \}. \end{aligned}$$

We denote by *d* the *Lévy metric* on ${\mathcal {M}}$, i.e., for all $\mu ,\nu \in {\mathcal {M}}$

$$\begin{aligned} d(\mu ,\nu ) := \inf \{\varepsilon >0 \,; \ \forall x\in {\mathbb {R}}, \ \nu (I_{x-\varepsilon })-\varepsilon \le \mu (I_x)\le \nu (I_{x+\varepsilon })+\varepsilon \}, \end{aligned}$$

where $I_x=(-\infty ,x]$ for $x\in {\mathbb {R}}$. We refer to Huber and Ronchetti (2009) for a complete account of the Lévy and related metrics and to Cont et al. (2010) for a discussion in a risk measure context. Let ${\mathcal {A}}\subset L^\infty$ be a law-invariant acceptance set. By law invariance, see Proposition 2.3, we can define a functional ${\mathcal {R}}_{\mathcal {A}}:{\mathcal {M}}^\infty \rightarrow {\mathbb {R}}$ by

$$\begin{aligned} {\mathcal {R}}_{\mathcal {A}}({\mathbb {P}}_X) := \rho _{\mathcal {A}}(X). \end{aligned}$$

By nonatomicity, for every $\mu \in {\mathcal {M}}^\infty$ there exists a sequence $(X_n)\subset L^\infty$ of i.i.d. random variables having $\mu$ as their common probability law. For each $n\in {\mathbb {N}}$ define the random measure $\mu _n:\Omega \rightarrow {\mathcal {M}}^\infty$ by

$$\begin{aligned} \mu _n(\omega ) := \frac{1}{n}\sum ^{n}_{i=1}\delta _{X_i(\omega )}, \end{aligned}$$

where $\delta$ denotes the standard Dirac measure. The historical estimator of ${\mathcal {R}}_{\mathcal {A}}(\mu )$ with sample size *n* is the function ${\mathcal {R}}_{\mathcal {A}}(\mu _n):\Omega \rightarrow {\mathbb {R}}$ defined by

$$\begin{aligned} {\mathcal {R}}_{\mathcal {A}}(\mu _n)(\omega ) := {\mathcal {R}}_{\mathcal {A}}(\mu _n(\omega )). \end{aligned}$$

For the remainder of the paper, we assume that ${\mathcal {R}}_{\mathcal {A}}(\mu _n)$ is measurable for every $n\in {\mathbb {N}}$.^1^ If ${\mathcal {A}}$ is convex, then historical estimators are strongly consistent in the sense that ${\mathcal {R}}_{\mathcal {A}}(\mu _n)\rightarrow {\mathcal {R}}_{\mathcal {A}}(\mu )$ almost surely. This follows, e.g., from Krätschmer et al. (2014, Theorem 2.6). The following definition of qualitative robustness captures the idea that, as long as a change in the law of the underlying data is small, the law of the corresponding historical estimator cannot change drastically.

### Definition 4.1

We say that ${\mathcal {R}}_{\mathcal {A}}$ is *(qualitatively) robust* on ${\mathcal {M}}^\infty$ if for all $\mu \in {\mathcal {M}}^\infty$ and $\varepsilon >0$ there exist $\delta >0$ and $n_0\in {\mathbb {N}}$ such that

$$\begin{aligned} d(\mu ,\nu )\le \delta \ \implies \ d({\mathbb {P}}_{{\mathcal {R}}_{\mathcal {A}}(\mu _n)},{\mathbb {P}}_{{\mathcal {R}}_{\mathcal {A}}(\nu _n)})\le \varepsilon \end{aligned}$$

for all $\nu \in {\mathcal {M}}^\infty$ and $n\ge n_0$.

A refined notion of qualitative robustness was proposed in Krätschmer et al. (2012) and further studied in Krätschmer et al. (2014). A critical observation in these papers is that two probability laws may possess a (very) different tail behavior but be rather close with respect to the Lévy metric (or, equivalently, with respect to any metric inducing the weak topology on ${\mathcal {M}}$ such as the Prohorov metric). As a result, establishing qualitative robustness does not imply that ${\mathcal {R}}_{\mathcal {A}}$ can sufficiently distinguish different tail profiles. The following refinement of the notion of qualitative robustness put forward in the above papers was meant to offer a way to overcome this limitation. For $p\in [1,\infty )$ define $\psi _p(x)=\frac{1}{p}\left| x\right| ^p$, $x\in {\mathbb {R}}$, and recall that a set ${\mathcal {N}}\subset {\mathcal {M}}$ is said to be *uniformly p-integrating* if it satisfies

$$\begin{aligned} \lim _{c\rightarrow \infty }\sup _{\mu \in {\mathcal {N}}}\int _{\{\psi _p\ge c\}}\psi _p(x)d\mu (x)=0. \end{aligned}$$

The desired refinement is obtained by adding a suitable tail sensitive component to the Lévy metric.

### Definition 4.2

Let $p\in [1,\infty )$. We say that ${\mathcal {R}}_{\mathcal {A}}$ is *(qualitatively) p-robust* on ${\mathcal {M}}^\infty$ if for all uniformly *p*-integrating sets ${\mathcal {N}}\subset {\mathcal {M}}^\infty$, $\mu \in {\mathcal {N}}$, and $\varepsilon >0$ there exist $\delta >0$ and $n_0\in {\mathbb {N}}$ such that

$$\begin{aligned} d(\mu ,\nu )+\left| \int _{\mathbb {R}}\psi _p(x)d\mu (x)-\int _{\mathbb {R}}\psi _p(x)d\nu (x)\right| \le \delta \ \implies \ d({\mathbb {P}}_{{\mathcal {R}}_{\mathcal {A}}(\mu _n)},{\mathbb {P}}_{{\mathcal {R}}_{\mathcal {A}}(\nu _n)})\le \varepsilon \end{aligned}$$

for all $\nu \in {\mathcal {N}}$ and $n\ge n_0$.

As suggested by Krätschmer et al. (2014), one can use the index *p* in the preceding definition in order to quantify the degree of qualitative robustness of historical estimators. We set $\inf \emptyset :=\infty$ and $\frac{1}{\infty }:=0$.

### Definition 4.3

Let ${\mathcal {A}}\subset L^\infty$ be a law-invariant acceptance set. The *index of qualitative robustness* of $\rho _{\mathcal {A}}$ is the number in [0, 1] defined by

$$\begin{aligned} {\mathrm{iqr}}(\rho _{\mathcal {A}}) := \frac{1}{\inf \{p\in [1,\infty ) \,; \ {\mathcal {R}}_{\mathcal {A}} \text{ is } p\text{-robust } \text{ on } {\mathcal {M}}^\infty \}}. \end{aligned}$$

It turns out that the degree of qualitative robustness of a risk measure is intimately related to the existence of finite extensions. As a result, as opposed to trying determine the index of qualitative robustness by applying its definition, we can equivalently focus on the index of finiteness, which is more direct and operationally simpler to compute.

### Theorem 4.4

(Koch-Medina and Munari (2014, Theorem 4.3), Krätschmer et al. (2014, Theorem 2.16)). Let ${\mathcal {A}}\subset L^\infty$ be a law-invariant convex acceptance set and take $p\in [1,\infty )$. The following are equivalent:

- $\rho _{\mathcal {A}}$ can be extended to a finite-valued (hence, continuous) cash-additive risk measure on $L^p$.
- ${\mathrm{cl}}_p({\mathcal {A}})$ has nonempty interior in $L^p$.
- ${\mathcal {R}}_{\mathcal {A}}$ is *p*-robust on ${\mathcal {M}}^\infty$.

Moreover, $${\mathrm{iqr}}(\rho _{\mathcal {A}})={\mathrm{fin}}(\rho _{\mathcal {A}})^{-1}$$.

### Remark 4.5

The index of qualitative robustness is defined in terms of a family of Orlicz functions, namely power functions associated with $L^p$ norms. In principle, one could take a different family of Orlicz functions to obtain a different definition of the index. Clearly, to define an index that allows to rank risk measures in a “total” way, one needs a continuum of embedded spaces. The family of $L^p$ spaces is clearly the natural choice. Another possibility is to take a continuum of Orlicz spaces linking $L^\infty$ with $L^1$, potentially indexed by a parameter appearing in the corresponding Orlicz functions. In the general case, the question would of course be that of selecting a continuum of spaces that are meaningful for the targeted application. Incidentally, note that the embedding between two general Orlicz spaces is a nontrivial problem but can be characterized under suitable assumptions; see, e.g., Edgar and Sucheston (1992, Proposition 2.2.1).

## Utility-based risk measures

We can now exploit the link between the index of qualitative robustness and the index of continuity to characterize the index of qualitative robustness of risk measures based on utility functions. Throughout the entire section, we fix a nonconstant, nondecreasing, right continuous, and concave function

$$\begin{aligned} u:{\mathbb {R}}\rightarrow [-\infty ,\infty ). \end{aligned}$$

Note that neither strict concavity nor differentiability of *u* is required. The function *u* is interpreted as a classical von Neumann-Morgenstern utility function. For any $\alpha \in {\mathbb {R}}$ such that $u(x)\ge \alpha$ for some $x\in {\mathbb {R}}$ we consider the law-invariant and convex acceptance set

$$\begin{aligned} {\mathcal {A}}_{u,\alpha } := \{X\in L^\infty \,; \ {\mathbb {E}}[u(X)]\ge \alpha \}. \end{aligned}$$

We denote by $\rho _{u,\alpha }:L^\infty \rightarrow {\mathbb {R}}$ the corresponding cash-additive risk measure, i.e.

$$\begin{aligned} \rho _{u,\alpha }(X) := \rho _{{\mathcal {A}}_{u,\alpha }}(X) = \inf \{m\in {\mathbb {R}}\,; \ {\mathbb {E}}[u(X+m)]\ge \alpha \}. \end{aligned}$$

Our objective is to provide an explicit formula for the index of qualitative robustness of $\rho _{u,\alpha }$. We start by highlighting that the expectation ${\mathbb {E}}[u(X)]$ is well defined for every random variable $X\in L^1$ and collecting a few properties of the expected utility functional ${\mathbb {E}}_u:L^1\rightarrow [-\infty ,\infty ]$ defined by ${\mathbb {E}}_u(X) := {\mathbb {E}}[u(X)]$.

### Proposition 5.1

For every $X\in L^1$ the expectation ${\mathbb {E}}[u(X)]$ is well defined and satisfies ${\mathbb {E}}[u(X)]<\infty$. Moreover, the following properties hold:

(i) ${\mathbb {E}}_u$ is nondecreasing, i.e., for all $X,Y\in L^1$
  $$\begin{aligned} Y\ge X \ \implies \ {\mathbb {E}}[u(Y)]\ge {\mathbb {E}}[u(X)]. \end{aligned}$$
(ii) ${\mathbb {E}}_u$ is concave, i.e., for all $X,Y\in L^1$ and $\lambda \in [0,1]$
  $$\begin{aligned} {\mathbb {E}}[u(\lambda X+(1-\lambda )Y)] \ge \lambda {\mathbb {E}}[u(X)]+(1-\lambda ){\mathbb {E}}[u(Y)]. \end{aligned}$$
(iii) ${\mathbb {E}}_u$ is upper semicontinuous, i.e., for all $(X_n)\subset L^1$ and $X\in L^1$
  $$\begin{aligned} X_n\rightarrow X \text{ in } L^1 \ \implies \ {\mathbb {E}}[u(X)] \ge \limsup _{n\rightarrow \infty }{\mathbb {E}}[u(X_n)]. \end{aligned}$$
(iv) ${\mathbb {E}}_u$ is law invariant, i.e., for all $X,Y\in L^1$
  $$\begin{aligned} {\mathbb {P}}_X={\mathbb {P}}_Y \ \implies \ {\mathbb {E}}[u(X)]={\mathbb {E}}[u(Y)]. \end{aligned}$$

### Proof

Note that, by concavity of *u*, there exist $a>0$ and $b\in {\mathbb {R}}$ such that $u(x)\le ax+b$ for every $x\in {\mathbb {R}}$. Hence, ${\mathbb {E}}[\max \{u(X),0\}] \le a{\mathbb {E}}[|X|]+|b| < \infty$ for every $X\in L^1$, showing that ${\mathbb {E}}[u(X)]$ is well defined and satisfies ${\mathbb {E}}[u(X)]<\infty$. As *u* is nondecreasing and concave, it follows that ${\mathbb {E}}_u$ is also nondecreasing and concave. Moreover, it is clear that ${\mathbb {E}}_u$ is law invariant. To show upper semicontinuity, let $(X_n)$ be a sequence of elements in $L^1$ converging to some $X\in L^1$. Without loss of generality, suppose that $\left\| X_n-X\right\| _1\le 2^{-n}$ for every $n\in {\mathbb {N}}$. We can define

$$\begin{aligned} Y_n = \sum ^{\infty }_{k=n}\left| X_k-X\right| \in L^1 \end{aligned}$$

and set $Z_n=X+Y_n\in L^1$ for every $n\in {\mathbb {N}}$. Note that $Z_n\ge X_n$ and, thus, ${\mathbb {E}}[u(Z_n)] \ge {\mathbb {E}}[u(X_n)]$ for every $n\in {\mathbb {N}}$. By construction, $Z_n\downarrow X$ almost surely. As *u* is right continuous, it follows that $u(Z_n)\downarrow u(X)$ almost surely as well. As ${\mathbb {E}}[u(Z_1)]<\infty$, it follows from the version of Fatou’s lemma in Shiryaev (2008, Theorem 2) that ${\mathbb {E}}[u(X)] \ge \limsup _{n\rightarrow \infty }{\mathbb {E}}[u(Z_n)] \ge \limsup _{n\rightarrow \infty }{\mathbb {E}}[u(X_n)]$. This shows that ${\mathbb {E}}_u$ is upper semicontinuous and concludes the proof. $\square$

For $p\in [1,\infty )$, the superlevel sets in $L^p$ of the expected utility functional are defined for every $\alpha \in {\mathbb {R}}$ by

$$\begin{aligned} {\mathcal {A}}^p_{u,\alpha } := \{X\in L^p \,; \ {\mathbb {E}}[u(X)]\ge \alpha \}. \end{aligned}$$

The next proposition collects their main properties.

### Proposition 5.2

Let $p\in [1,\infty )$ and take $\alpha \in {\mathbb {R}}$ such that $u(x)\ge \alpha$ for some $x\in {\mathbb {R}}$. The set ${\mathcal {A}}^p_{u,\alpha }$ is a law-invariant, convex, $L^p$-closed, acceptance set. Moreover, ${\mathcal {A}}^p_{u,\alpha }={\mathrm{cl}}_p({\mathcal {A}}_{u,\alpha })$.

### Proof

Note that ${\mathcal {A}}^p_{u,\alpha }$ is nonempty by assumption on $\alpha$. It follows from the properties of the functional ${\mathbb {E}}_u$ recorded in Proposition 5.1 that ${\mathcal {A}}^p_{u,\alpha }$ is law invariant, convex, closed, and monotone. To show that it is a strict subset of $L^p$, it suffices to observe that $u(x)\rightarrow -\infty$ as $x\rightarrow -\infty$ by concavity of *u*. Now, note that ${\mathcal {A}}_{u,\alpha }\subset {\mathcal {A}}^p_{u,\alpha }$. By closedness, it then remains to show that ${\mathcal {A}}^p_{u,\alpha }\subset {\mathrm{cl}}_p({\mathcal {A}}_{u,\alpha })$. To this effect, take $X\in {\mathcal {A}}^p_{u,\alpha }$ and assume first that $u(x)\le \alpha$ for every $x\in {\mathbb {R}}$. In this case, set $m=\inf \{x\in {\mathbb {R}}\,; \ u(x)=\alpha \}$. Note that $u(m)=\alpha$ by right continuity. Since ${\mathbb {E}}[u(X)]\ge \alpha$, we must have $X\ge m$. Set for every $n\in {\mathbb {N}}$

$$\begin{aligned} X_n = X1_{\{X\le n\}}+ m1_{\{X>n\}} \in L^\infty . \end{aligned}$$

It is immediate to see that $X_n\rightarrow X$ in $L^p$ and ${\mathbb {E}}[u(X_n)]\ge u(m)=\alpha$ for every $n\in {\mathbb {N}}$. Next, suppose that $u(x)>\alpha$ for some $x\in {\mathbb {R}}$. In this case, set $X_\lambda =\lambda X+(1-\lambda )x$ for $\lambda \in (0,1)$ and observe that $X_\lambda \rightarrow X$ in $L^p$ as $\lambda \rightarrow 1$. By concavity of *u*, we have for every $\lambda \in (0,1)$

$$\begin{aligned} {\mathbb {E}}[u(X_\lambda )] \ge \lambda {\mathbb {E}}[u(X)]+(1-\lambda )u(x) > \alpha . \end{aligned}$$

This shows that we can assume without loss of generality that ${\mathbb {E}}[u(X)]>\alpha$. In this case, set for $n\in {\mathbb {N}}$

$$\begin{aligned} X_n = X1_{\{|X|\le n\}}-n1_{\{X<-n\}}+n1_{\{X>n\}} \in L^\infty . \end{aligned}$$

Note that $u(X_n)\rightarrow u(X)$ almost surely and for *n* large enough $u(-n)<0$ and $u(n)\ge \alpha$, so that

$$\begin{aligned} |u(X_n)|&= |u(X)|1_{\{|X|\le n\}}-u(-n)1_{\{X<-n\}}+|u(n)|1_{\{X>n\}} \\&\le |u(X)|1_{\{X\le n\}}+\max \{|u(X)|,|\alpha |\}1_{\{X>n\}} \\&\le \max \{|u(X)|,|\alpha |\}. \end{aligned}$$

Note that $|u(X)|\in L^1$ by Proposition 5.1. Hence, the dominated convergence theorem delivers ${\mathbb {E}}[u(X_n)]\rightarrow {\mathbb {E}}[u(X)]$, implying that ${\mathbb {E}}[u(X_n)]>\alpha$ for *n* large enough. This concludes the proof. $\square$

### Remark 5.3

The equality ${\mathcal {A}}^p_{u,\alpha }={\mathrm{cl}}_p({\mathcal {A}}_{u,\alpha })$ also follows from a general result about convex law-invariant sets; see Bellini et al. (2021, Corollary 4.3). The above proof exploits the particular structure of the sets ${\mathcal {A}}^p_{u,\alpha }$.

Our next proposition links the finiteness and continuity of the expected utility functional with the existence of finite and continuous extensions of the corresponding utility-based risk measure. This result will play a key role in the characterization of the index of qualitative robustness for utility-based risk measures. We use the notation $u_\infty :=\sup \{u(x) \,; \ x\in {\mathbb {R}}\}$ and follow the convention $\frac{1}{0}:=\infty$.

### Proposition 5.4

Let $p\in [1,\infty )$ and take $\alpha <u_\infty$. The following statements are equivalent:

- $\rho _{u,\alpha }$ can be extended to a finite-valued (hence, continuous) cash-additive risk measure on $L^p$.
- ${\mathbb {E}}_u$ is finite valued and continuous on $L^p$.
- ${\mathcal {A}}^p_{u,\alpha }$ has nonempty interior in $L^p$.
- $$\limsup _{x\rightarrow \infty }\frac{x^p}{u(-x)}<0$$.

### Proof

Note that $\{X\in L^p \,; \ {\mathbb {E}}[u(X)]>\alpha \}$ is a nonempty open set whenever ${\mathbb {E}}_u$ is continuous on $L^p$. This shows that *(b)* implies *(c)*. Now, assume that *(c)* holds and let $X\in L^p$ be an interior point of ${\mathcal {A}}^p_{u,\alpha }$. By density of $L^\infty$ in $L^p$, we can assume that $X\in L^\infty$. In particular, $u(\Vert X\Vert _\infty )\ge \alpha$. If *(d)* does not hold, then $\limsup _{x\rightarrow \infty }\frac{x^p}{u(-x)}=0$. This implies that for every $m>0$

$$\begin{aligned} \sup _{x\ge m}\frac{x^p}{u(-x)} = 0. \end{aligned}$$

Hence, for every $r>0$ we can find $m>r$ sufficiently large to satisfy

$$\begin{aligned} 0< \frac{m^p}{u(\Vert X\Vert _\infty )-u(\Vert X\Vert _\infty -m)} < \frac{r^p}{u(\Vert X\Vert _\infty )-\alpha }, \end{aligned}$$

where the right-hand side inequality is trivial if $u(\Vert X\Vert _\infty )=\alpha$. Rearranging yields

$$\begin{aligned} 0 \le \frac{u(\Vert X\Vert _\infty )-\alpha }{u(\Vert X\Vert _\infty )-u(\Vert X\Vert _\infty -m)}< \frac{r^p}{m^p} < 1. \end{aligned}$$

As a consequence, we find $\lambda \in (0,1)$ such that

$$\begin{aligned} \frac{u(\Vert X\Vert _\infty )-\alpha }{u(\Vert X\Vert _\infty )-u(\Vert X\Vert _\infty -m)}< \lambda < \frac{r^p}{m^p}. \end{aligned}$$

In particular, note that $\lambda m^p<r^p$ and

$$\begin{aligned} \lambda u(\Vert X\Vert _\infty -m) + (1-\lambda )u(\Vert X\Vert _\infty ) < \alpha . \end{aligned}$$

By nonatomicity, we find $E\in {\mathcal {F}}$ with ${\mathbb {P}}(E)=\lambda$. Now, set $Z=X-m1_E\in L^p$. Clearly,

$$\begin{aligned} {\mathbb {E}}[u(Z)] = {\mathbb {E}}[u(X-m1_E)] \le \lambda u(\Vert X\Vert _\infty -m)+(1-\lambda )u(\Vert X\Vert _\infty ) < \alpha . \end{aligned}$$

Moreover, $\left\| Z-X\right\| ^p_p=\lambda m^p<r^p$. This shows that ${\mathcal {A}}^p_{u,\alpha }$ contains no $L^p$-neighborhood of *X*. As this goes against our assumption, we conclude that *(d)* must hold. Next, assume that *(d)* holds so that we find $\varepsilon >0$ and $m>0$ such that $u(-m)<0$ and

$$\begin{aligned} \sup _{x\ge m}\frac{x^p}{u(-x)} \le -\varepsilon . \end{aligned}$$

This yields $x^p\ge -\varepsilon u(-x)$ whenever $x\ge m$. As a consequence,

$$\begin{aligned} {\mathbb {E}}[u(X)]&= {\mathbb {E}}[u(X)1_{\{X<-m\}}]+{\mathbb {E}}[u(X)1_{\{X\ge -m\}}] \\&\ge -\frac{1}{\varepsilon }{\mathbb {E}}[(-X)^p1_{\{X<-m\}}]+{\mathbb {P}}(X\ge -m)u(-m) \\&\ge -\frac{1}{\varepsilon }\left\| X\right\| ^p_p+{\mathbb {P}}(X\ge -m)u(-m) > -\infty \end{aligned}$$

for every $X\in L^p$. Hence, by Proposition 5.1, the map ${\mathbb {E}}_u$ is finite valued on $L^p$. The monotonicity of ${\mathbb {E}}_u$ implies, by Proposition 3.1, that ${\mathbb {E}}_u$ is also continuous on $L^p$, proving *(b)*. To conclude the proof, it remains to observe that *(a)* and *(c)* are equivalent by Theorem 3.3 and Proposition 5.2. $\square$

### Remark 5.5

For many standard utility functions the limsup in the preceding proposition can be replaced by a limit; see Example 5.8. However, for a general utility function, convergence may fail and the limsup may therefore be necessary, as shown in the following example. Let $p\in (1,\infty )$. We construct a strictly-increasing sequence $(x_n)\subset (0,\infty )$ and a strictly-increasing convex function $f:[0,\infty )\rightarrow [0,\infty )$ such that for every $n\in {\mathbb {N}}$

5.1 $$\begin{aligned} \frac{x^p_{2n-1}}{f(x_{2n-1})}\ge 2, \ \ \ \ \frac{x^p_{2n}}{f(x_{2n})}\le 1. \end{aligned}$$

It will therefore suffice to assume that $u(-x)=-f(x)$ for every $x\ge 0$ to see that $\frac{x^p}{u(-x)}$ does not admit a limit for $x\rightarrow \infty$. The function *f* is a piecewise linear function of the form

$$\begin{aligned} f(x)= {\left\{ \begin{array}{ll} \alpha _1x+\beta _1 &{} \text{ if } \ x\in [0,x_1],\\ \alpha _{2n+1}x+\beta _{2n+1} &{} \text{ if } \ x\in (x_{2n-1},x_{2n+1}], \ n\in {\mathbb {N}}. \end{array}\right. } \end{aligned}$$

We ensure strict monotonicity and convexity by assuming for every $n\in {\mathbb {N}}$ that $0<\alpha _{2n-1}<\alpha _{2n+1}$ and

$$\begin{aligned} \beta _{2n+1}=\alpha _{2n-1}x_{2n-1}+\beta _{2n-1}-\alpha _{2n+1}x_{2n-1}. \end{aligned}$$

It remains to fix the sequences $(x_n)$ and $(\alpha _{2n-1})$ and the coefficient $\beta _1$ in such a way that (5.1) holds. We proceed by recursion. In a first step, we ensure (5.1) for $n=1$. To this effect, set $x_1=1$ and $x_2=2$. Moreover, define $\alpha _1=\frac{1}{2}$ and $\beta _1=0$. Finally, take $\alpha _3\ge x^p_2-\alpha _1x_1-\beta _1$. It is easy to verify that

$$\begin{aligned} \frac{x^p_1}{f(x_1)}=\frac{x^p_1}{\alpha _1x_1+\beta _1}=2, \ \ \ \ \frac{x^p_2}{f(x_2)}=\frac{x^p_2}{\alpha _3x_2+\beta _3}= \frac{x^p_2}{\alpha _3(x_2-x_1)+\alpha _1x_1+\beta _1}\le 1. \end{aligned}$$

Now, assume that we have determined $x_{2n-1}$ and $x_{2n}$ as well as $\alpha _{2n-1}$ and $\alpha _{2n+1}$ so that (5.1) holds for some $n\in {\mathbb {N}}$. Take $x_{2n+1}>x_{2n}$ large enough to satisfy $x^p_{2n+1}\ge 2(\alpha _{2n+1}x_{2n+1}+\beta _{2n+1})$. Moreover, set $x_{2n+2}=x_{2n+1}+1$ and take $\alpha _{2n+3}\ge x_{2n+2}^p-\alpha _{2n+1}x_{2n+1}-\beta _{2n+1}$. It follows that

$$\begin{aligned}&\frac{x^p_{2n+1}}{f(x_{2n+1})}=\frac{x^p_{2n+1}}{\alpha _{2n+1}x_{2n+1}+\beta _{2n+1}}\ge 2, \\&\frac{x^p_{2n+2}}{f(x_{2n+2})}=\frac{x^p_{2n+2}}{\alpha _{2n+3}x_{2n+2}+\beta _{2n+3}}= \frac{x^p_{2n+2}}{\alpha _{2n+3}(x_{2n+2}-x_{2n+1})+\alpha _{2n+1}x_{2n+1}+\beta _{2n+1}}\le 1. \end{aligned}$$

This shows that (5.1) holds for $n+1$, concluding the construction by recursion.

The previous result can be exploited to derive our desired formula for the index of qualitative robustness for utility-based risk measures. In the interesting situations, the index is determined by the decay behavior of the utility function for large losses.

### Theorem 5.6

Let $\alpha \in {\mathbb {R}}$ satisfy $u(x)\ge \alpha$ for some $x\in {\mathbb {R}}$. The following statements hold:

(i) If $u(x)\le \alpha$ for every $x\in {\mathbb {R}}$, then ${\mathrm{iqr}}(\rho _{u,\alpha })=0$.
(ii) If $u(x)>\alpha$ for some $x\in {\mathbb {R}}$, then
  $$\begin{aligned} {\mathrm{iqr}}(\rho _{u,\alpha }) = \frac{1}{{\mathrm{fin}}(\rho _{u,\alpha })} = \frac{1}{\inf \{p\in [1,\infty ) \,; \ \limsup _{x\rightarrow \infty }\frac{x^p}{u(-x)}<0\}}. \end{aligned}$$

### Proof

If $u(x)\le \alpha$ for every $x\in {\mathbb {R}}$, then for $p\in [1,\infty )$ we have ${\mathcal {A}}^p_{u,\alpha }=\{X\in L^p \,; \ {\mathbb {E}}[u(X)]=\alpha \}$. This set has empty interior in $L^p$. Indeed, for every $X\in {\mathcal {A}}^p_{u,\alpha }$ it is enough to take $m\in {\mathbb {R}}$ with $u(m)<\alpha$ and a sequence $(E_n)\subset {\mathcal {F}}$ such that ${\mathbb {P}}(E_n)=\frac{1}{n}$ for every $n\in {\mathbb {N}}$, which exists by nonatomicity, and set $X_n=1_{E_n}m+1_{E^c_n}X\in L^p$ for every $n\in {\mathbb {N}}$. Then, $X_n\rightarrow X$ in $L^p$ but ${\mathbb {E}}[u(X_n)]<{\mathbb {E}}[u(X)]=\alpha$ for every $n\in {\mathbb {N}}$. This yields ${\mathrm{fin}}(\rho _{u,\alpha })=\infty$ by Theorem 3.3. Otherwise, we infer from Proposition 5.4 that

$$\begin{aligned} {\mathrm{fin}}(\rho _{u,\alpha }) = \inf \left\{ p\in [1,\infty ) \,; \ \limsup _{x\rightarrow \infty }\frac{x^p}{u(-x)}<0\right\} . \end{aligned}$$

The desired statement is now a direct consequence of Theorem 4.4. $\square$

### Remark 5.7

(i) The preceding theorem extends a result in Koch-Medina and Munari (2014), which was obtained under the assumption that *u* is bounded from above and that $\frac{x^p}{u(-x)}$ admits a limit for $x\rightarrow \infty$ for every $p\in [1,\infty )$. Even though the asymptotic behavior of *u* at $\infty$ has no influence on the result itself, the assumption was used to prove a technical preliminary step. As such, the result for general, possibly unbounded above, utility functions cannot be derived from the result in that paper. The strategy we pursued here is more direct and works for every *u*.

(ii) The condition $u(x)\ge \alpha$ for some $x\in {\mathbb {R}}$ ensures that ${\mathcal {A}}_{u,\alpha }\cap L^\infty$ is not empty and, hence, qualifies as an acceptance set by Proposition 5.2.

We conclude by determining the index of qualitative robustness for a variety of concrete utility functions. The main message arising from the preceding theorem is that only the “tail” behavior of *u*, i.e., the behavior at $-\infty$, matters to compute the index of qualitative robustness. This is why, for our purposes, we can distinguish utility functions based on their “tail” behavior. In what follows we always choose $\alpha$ so as to satisfy $\alpha <u(x)$ for some $x\in {\mathbb {R}}$.

1. *Power tail* If $q\in [1,\infty )$ and *u*(*x*) is asymptotic to $-|x|^q$ for $x\rightarrow -\infty$, then
  $$\begin{aligned} \lim _{x\rightarrow \infty }\frac{x^p}{u(-x)}= {\left\{ \begin{array}{ll} -\infty &{} \text{ if } \ p>q,\\ -1 &{} \text{ if } \ p=q,\\ 0 &{} \text{ if } \ p<q, \end{array}\right. } \end{aligned}$$
  for every $p\in [1,\infty )$. As a result, ${\mathrm{iqr}}(\rho _{u,\alpha })=\frac{1}{q}$.
2. *Exponential tail* If $\gamma >0$ and *u*(*x*) is asymptotic to $-e^{-\gamma x}$ for $x\rightarrow -\infty$, then
  $$\begin{aligned} \lim _{x\rightarrow \infty }\frac{x^p}{u(-x)} = 0 \end{aligned}$$
  for every $p\in [1,\infty )$. As a result, ${\mathrm{iqr}}(\rho _{u,\alpha })=0$.

The following examples are special instances of the preceding results. It should be noted that the well-known entropic risk measure, i.e., the risk measure corresponding to an exponential utility function, exhibits a poor index of qualitative robustness compared to other cases. We denote by $\mathrm{NA}$ the case where the index of qualitative robustness cannot be computed because the underlying risk measure is degenerate in the sense that ${\mathcal {A}}_{u,\alpha }=\emptyset$ and, hence, $\rho _{u,\alpha }\equiv \infty$. The first two examples are discussed, e.g., in Ben-Tal and Teboulle (2006). The other three examples are discussed, e.g., in Henderson and Hobson (2009).

### Example 5.8

(i) *Gain-loss linear utility* Let $\gamma _1>\gamma _2\ge 0$ and define the utility function

$$\begin{aligned} u(x)=\gamma _1 x1_{(-\infty ,0)}(x)+\gamma _2 x1_{[0,\infty )}(x), \ \ \ x\in {\mathbb {R}}. \end{aligned}$$

For every $\alpha \in {\mathbb {R}}$ we have

$$\begin{aligned} {\mathrm{iqr}}(\rho _{u,\alpha })= {\left\{ \begin{array}{ll} 1 &{} \text{ if } \ \gamma _2>0 \text{ and } \alpha \in {\mathbb {R}},\\ 1 &{} \text{ if } \ \gamma _2=0 \text{ and } \alpha <0,\\ 0 &{} \text{ if } \ \gamma _2=0 \text{ and } \alpha =0,\\ \mathrm{NA} &{} \text{ if } \ \gamma _2=0 \text{ and } \alpha >0. \end{array}\right. } \end{aligned}$$

(ii) *Quadratic utility* Let $\gamma >0$ and define the utility function

$$\begin{aligned} u(x)=(x-\gamma x^2)1_{(-\infty ,1/2\gamma )}(x)+\frac{1}{4\gamma }1_{[1/2\gamma ,\infty )}(x), \ \ \ x\in {\mathbb {R}}. \end{aligned}$$

For every $\alpha \in {\mathbb {R}}$ we have

$$\begin{aligned} {\mathrm{iqr}}(\rho _{u,\alpha })= {\left\{ \begin{array}{ll} \frac{1}{2} &{} \text{ if } \ \alpha <\frac{1}{4\gamma },\\ 0 &{} \text{ if } \ \alpha =\frac{1}{4\gamma },\\ \mathrm{NA} &{} \text{ if } \ \alpha >\frac{1}{4\gamma }. \end{array}\right. } \end{aligned}$$

(iii) *Dampened quadratic utility* Let $\gamma >0$ and define the utility function

$$\begin{aligned} u(x)=\frac{1}{\gamma }(1+\gamma x-\sqrt{1+\gamma ^2x^2}), \ \ \ x\in {\mathbb {R}}. \end{aligned}$$

For every $\alpha \in {\mathbb {R}}$ we have

$$\begin{aligned} {\mathrm{iqr}}(\rho _{u,\alpha })= {\left\{ \begin{array}{ll} 1 &{} \text{ if } \ \alpha <\gamma ,\\ \mathrm{NA} &{} \text{ if } \ \alpha \ge \gamma . \end{array}\right. } \end{aligned}$$

(iv) *Exponential utility* Let $\gamma >0$ and define the utility function

$$\begin{aligned} u(x)=\frac{1}{\gamma }(1-e^{-\gamma x}), \ \ \ x\in {\mathbb {R}}. \end{aligned}$$

For every $\alpha \in {\mathbb {R}}$ we have

$$\begin{aligned} {\mathrm{iqr}}(\rho _{u,\alpha })= {\left\{ \begin{array}{ll} 0 &{} \text{ if } \ \alpha <\frac{1}{\gamma },\\ \mathrm{NA} &{} \text{ if } \ \alpha \ge \frac{1}{\gamma }. \end{array}\right. } \end{aligned}$$

(v) *Amplified exponential utility* Let $\gamma >0$ and define the utility function

$$\begin{aligned} u(x)=\frac{1}{\gamma }(1+x-e^{-\gamma x}), \ \ \ x\in {\mathbb {R}}. \end{aligned}$$

For every $\alpha \in {\mathbb {R}}$ we have ${\mathrm{iqr}}(\rho _{u,\alpha })=0$.

## Footnotes

1 This is always possible provided $(\Omega ,{\mathcal {F}},{\mathbb {P}})$ is chosen appropriately as done, e.g., in Krätschmer et al. (2014). In that setting, measurability follows by composition from the measurability of the maps $\mu _n$ and ${\mathcal {R}}_{\mathcal {A}}$, where ${\mathcal {M}}^\infty$ is equipped with the Borel $\sigma$-field induced by the Lévy metric. We refer to Dudley (2004) and Weber (2006) for details about measurability of $\mu _n$ and ${\mathcal {R}}_{\mathcal {A}}$, respectively. Note that the particular choice of $(\Omega ,{\mathcal {F}},{\mathbb {P}})$ plays no critical role in our statistical inference problem as we focus exclusively on distribution functions.
